# Beyond the Usual Suspects: Cytomegalovirus-Driven Hemophagocytic Lymphohistiocytosis (HLH) in a Previously Healthy Adult

**DOI:** 10.7759/cureus.110540

**Published:** 2026-06-09

**Authors:** Noor Eweis, Tripti Adhikari, Elizabeth Pionk, Graham Mcgee, Samar Mehyar

**Affiliations:** 1 Internal Medicine, McLaren Bay Region, Bay City, USA; 2 Infectious Diseases, McLaren Bay Region, Bay City, USA

**Keywords:** cytokine release storm, cytokine storm, cytomegalovirus (cmv), drug-induced liver injury (dili), fever of unknown origin (fuo), hemophagocytic lymphohistocytosis (hlh), immunocompetent host, macrophage activation syndrome (mas), secondary hlh, transaminitis-raised liver enzyme

## Abstract

Hemophagocytic lymphohistiocytosis (HLH) is a potentially life-threatening syndrome characterized by excessive immune activation and cytokine storm. While primary HLH is genetic and typically presents in childhood, secondary HLH is acquired and often triggered by malignancy, autoimmune disorders, or infections. Acute cytomegalovirus (CMV) infection is a known trigger, although it is rarely the primary cause in immunocompetent adults. We present the case of a healthy 33-year-old female presenting with three weeks of high-grade fever and flank pain following treatment for a presumed urinary tract infection. Despite multiple courses of antibiotics, her symptoms persisted. Evaluation revealed hepatitis, cytopenias, and splenomegaly. Testing confirmed acute CMV infection with a relatively low viral titer. However, the patient met the diagnostic criteria for HLH with a high H-score. She was successfully treated with antivirals, corticosteroids, and intravenous immunoglobulin (IVIG). This case highlights the necessity of considering HLH in the differential diagnosis of fever of unknown origin (FUO) and underscores that even mild CMV viremia can precipitate life-threatening HLH in immunocompetent adults.

## Introduction

Hemophagocytic lymphohistiocytosis (HLH) is a clinical syndrome characterized by a severe, exaggerated immune response that can be life-threatening if not recognized and treated early. HLH is predominantly observed in adults and is typically triggered by underlying malignancies, autoimmune disorders (often termed macrophage activation syndrome), or infections [1]. Among infectious triggers, viruses are the most common culprits, with Epstein-Barr virus (EBV) being the most frequently cited [2]. However, cytomegalovirus (CMV) has also been implicated in rare cases. While acute CMV infection in immunocompetent individuals is often asymptomatic or presents as a mild mononucleosis-like illness, it can occasionally precipitate the "cytokine storm" - a dysregulated immune response characterized by the release of excessive cytokines - characteristic of HLH [3]. This presentation is particularly challenging to diagnose in immunocompetent adults, as the viral symptoms may be dismissed as incidental.

We present the case of a previously healthy 33-year-old female with prolonged fever and end-organ dysfunction. Her workup revealed CMV-induced HLH, highlighting the diagnostic challenge of this condition and the importance of early immunomodulatory intervention in preventing fatal outcomes.

## Case presentation

A 33-year-old previously healthy female nurse presented to the hospital with a chief complaint of high-grade fever occurring intermittently for three weeks. Her symptoms were associated with one day of right upper quadrant (RUQ) and flank pain, nausea, and vomiting. Approximately three weeks prior to admission, the patient developed symptoms suggestive of a urinary tract infection (UTI). She was initially treated with a 10-day course of amoxicillin. Due to persistent fevers and symptoms, therapy was escalated to trimethoprim/sulfamethoxazole (TMP/SMX) and subsequently to ciprofloxacin. Despite these interventions, she remained intermittently febrile and symptomatic, prompting admission to our facility.

On physical examination, the patient was febrile and exhibited tenderness in the RUQ. Initial laboratory evaluation revealed significant liver dysfunction and elevated inflammatory markers (Table 1). Urinalysis demonstrated microscopic hematuria but was negative for leukocyte esterase and nitrites. Urine culture obtained on admission was negative. Notably, the patient endorsed a history of chronic microscopic hematuria noted on multiple prior urinalyses, suggesting that this was not an acute finding related to her current illness and was therefore irrelevant. A hepatitis viral panel and autoimmune workup were negative. Blood cultures were negative (x4). Computed tomography (CT) of the abdomen, ultrasound, and a HIDA (hepatobiliary iminodiacetic acid) scan were unremarkable on admission.

**Table 1:** Patient’s initial lab results ALP: alkaline phosphatase; ALT: alanine transaminase; AST: aspartate aminotransferase; CMV: cytomegalovirus; CRP: C-reactive protein; HLH: hemophagocytic lymphohistiocytosis; LDH: lactate dehydrogenase; PCR: polymerase chain reaction

Laboratory test	Patient value	Reference range
Hematology		
Hemoglobin	10	12.0-15.5 g/dL
Platelets	112	150-450 x 10³/µL
Reticulocyte count	4.78%	0.5-2.5%
Haptoglobin	<10 mg/dL	30-200 mg/dL
Liver function		
Alanine transaminase (ALT)	450 U/L	7-55 U/L
Aspartate aminotransferase (AST)	410 U/L	8-48 U/L
Alkaline phosphatase (ALP)	338 U/L	44-147 U/L
Direct bilirubin	1.05 mg/dL	0.1-0.3 mg/dL
Inflammatory and metabolic		
C-reactive protein (CRP)	4.7 mg/dL	<0.8 mg/dL
Lactate dehydrogenase (LDH)	616 U/L	140-280 U/L
Ferritin	549 ng/mL	13-150 ng/mL
Fibrinogen	199 mg/dL	200-400 mg/dL
Triglycerides	202 mg/dL	<150 mg/dL
Infectious and immunology		
CMV IgM antibody	Positive	Negative
CMV PCR (quantitative)	193 IU/mL	Not detected
Blood cultures (x4)	Negative	No growth
H-score (HLH probability)	200 (>88% probability)	N/A

The patient was started on empiric broad-spectrum antibiotics. However, she continued to spike daily fevers, predominantly in the evening, accompanied by nausea, vomiting, and dark urine. By hospital day 2, she developed worsening cytopenias and laboratory evidence of hemolysis, including elevated reticulocytes and LDH with low haptoglobin (Table 1). The Coombs test was negative, and complement C3/C4 levels were normal. Infectious workup returned positive for CMV IgM, with a quantitative PCR showing a low titer of 193 IU/ml. Due to such low levels of CMV in an immunocompetent host, it was not deemed to be an acute CMV infection or the culprit for her current presentation, but rather a reactivation of the virus in the setting of acute illness [2,4,5]. Other extensive infectious workup for fever of unknown origin (FUO) was negative, including human immunodeficiency virus (HIV), sexually transmitted diseases (STDs), infectious mononucleosis screen, Epstein-Barr virus deoxyribonucleic acid (EBV DNA), herpes simplex virus (HSV) plasma, polymerase chain reaction (PCR), Lyme, *Babesia*, *Anaplasma*, Rocky mountain spotted fever, *Bartonella*, and hepatitis E serologies. Fungitell and urine histoplasma antigen were negative as well. Triglyceride and ferritin levels were eventually checked and were elevated, while the fibrinogen level was low (Table 1).

**Figure 1:**
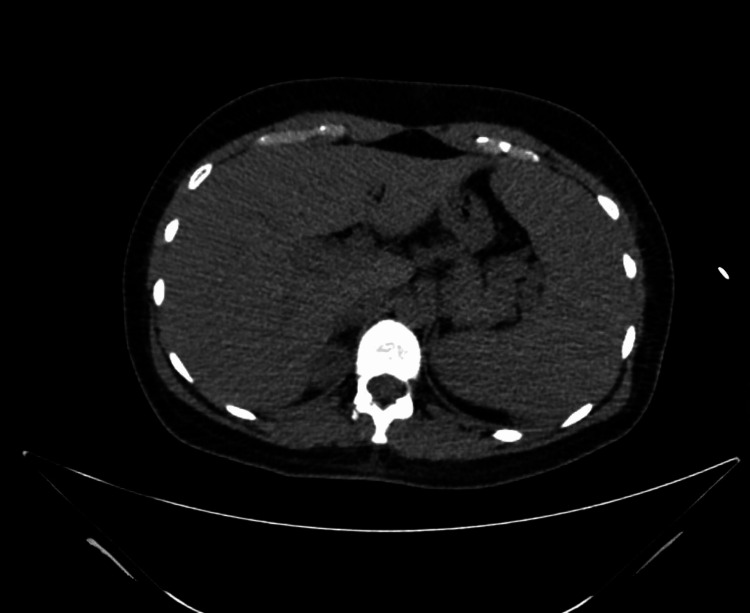
Patient’s non-contrast abdominal CT showing splenomegaly

Due to persistent liver enzyme elevation with a negative extensive workup, she underwent a CT-guided liver biopsy. The procedural CT revealed new, massive splenomegaly, which had not been present on admission imaging (Figure 1).

**Figure 2:**
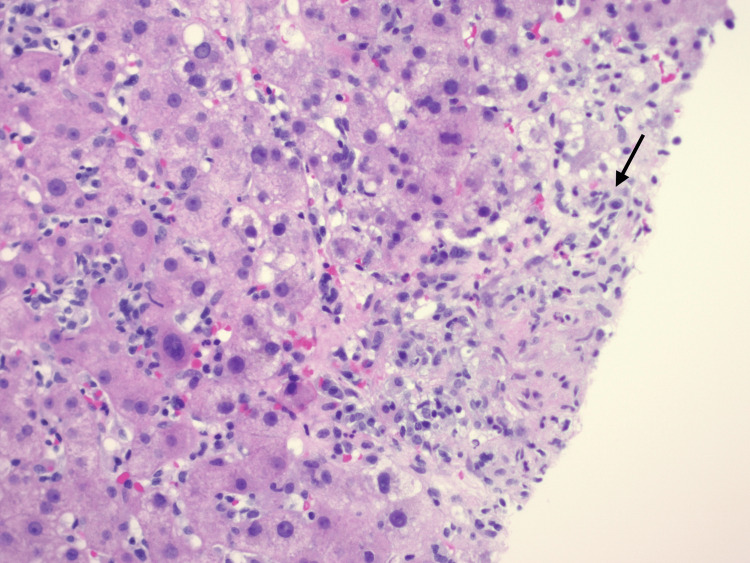
Liver biopsy specimen showing sinusoidal lymphocytosis, histiocytes engulfing platelets, and adjacent noncaseating granulomas (arrow) Patient’s pathology specimen; special thanks to Dr. Steven Reeves of McLaren Medical Group Pathology.

Empiric antibiotics were discontinued, and intravenous (IV) ganciclovir was initiated. While liver enzymes improved on antiviral therapy, fevers persisted. Given the constellation of fevers, splenomegaly, cytopenias, and hepatitis, suspicion for HLH was raised. Moreover, for early recognition, a scoring tool called H-score, which takes into account the above parameters to predict the probability of HLH, can be crucial. A cutoff value of 169 is used, corresponding to a sensitivity of 93% and specificity of 86% [6]. The H-score in our patient was calculated to be 200, indicating a >88% probability of HLH. A soluble CD25 (sCD25) level was obtained. Preliminary pathology from the liver biopsy reported hemophagocytosis. The patient was treated with a dose of empiric pulse steroids and intravenous immunoglobulin (IVIG). Following this intervention, she became afebrile for 24 hours. She was then transferred to a tertiary care center for expert hematological management. Subsequent final biopsy results showed sinusoidal lymphocytosis, Kupffer cell histiocytic aggregates, and focal areas of granulomatous inflammation (Figure 2). The findings were consistent with acute infectious hepatitis with histiocytic inflammation showing platelets and neutrophils within macrophage cytoplasm (hemophagocytosis). Soluble CD25 levels returned significantly elevated, confirming the diagnosis.

On the Infectious Disease follow-up 20 days after discharge from the higher center, she endorsed that her clinical condition had improved with the resolution of fever. We retested for CMV viral loads, CBC, and a complete metabolic panel. Results returned with undetectable CMV levels by PCR, improvement of hemoglobin by 2 g/dL, and resolution of transaminitis. Ferritin levels and CRP had also normalized.

## Discussion

This case of CMV-induced HLH in a young, immunocompetent adult underscores the critical importance of including HLH in the differential diagnosis of FUO, particularly in patients who deteriorate despite broad-spectrum antibiotic therapy. While HLH is increasingly recognized, its association with CMV in immunocompetent hosts remains a diagnostic pitfall, as a low-level viremia can be easily dismissed [4].

CMV is a well-established trigger for HLH, but its typical association is with immunocompromised states, such as post-transplant or HIV infection [2]. Our case aligns with a growing yet under-recognized body of literature documenting CMV-associated HLH in otherwise healthy adults [7,8]. A key diagnostic hurdle, as illustrated here, is the interpretation of low-titer CMV viremia (193 IU/mL). In contrast to immunocompromised patients, where high viral loads are common, immunocompetent hosts often present with modest viremia, leading to its erroneous classification as an incidental finding [4,8].

The histological confirmation of hemophagocytosis was paramount, yet the negative CMV immunohistochemistry (IHC) on liver biopsy initially introduced diagnostic uncertainty. This discordance is not uncommon and serves as a critical teaching point. While tissue IHC for CMV has a reported sensitivity of 78-93% [5], a negative result does not exclude infection, particularly in cases where the viral burden is focal or low. Previous case series have noted similar discrepancies, where systemic evidence of CMV (serum PCR) was definitive despite negative tissue staining [9]. Although tissue PCR is the gold standard for localized detection, it was unavailable in our case. The diagnosis, therefore, rested on synthesizing multiple data points: the characteristic histology of HLH, supportive serum CMV PCR, the exclusion of alternative etiologies (autoimmune hepatitis, drug-induced liver injury (DILI), and malignancy), and the dramatic clinical response to anti-CMV and immunomodulatory therapy. This holistic approach is emphasized in recent HLH diagnostic guidelines, which advocate for treating the clinical syndrome rather than relying on a single pathognomonic test [10].

Our patient unequivocally met six of the eight HLH-2004 diagnostic criteria (fever, splenomegaly, bi-lineage cytopenias, hypofibrinogenemia, hypertriglyceridemia, markedly elevated ferritin, and elevated sCD25), with histologic confirmation [6,10]. This demonstrates the robustness of these criteria even in atypical triggers like community-acquired CMV. However, our case also highlights a potential limitation. As noted in studies of adult-onset HLH, the HLH-2004 criteria were originally developed for pediatric familial forms and may not fully capture the subtler presentations sometimes seen in adult secondary HLH [11]. Nonetheless, the severity of our patient's presentation left little diagnostic ambiguity.

The pathophysiology involves a dysregulated immune response where persistent antigenic stimulation (CMV in our case) leads to uncontrolled activation of T-cells and macrophages, resulting in a life-threatening cytokine storm [3]. This mechanism is consistent across triggers, but the treatment strategy must be dual: eradicating the inciting antigen and suppressing the hyperactive immune system [10]. Our successful use of ganciclovir alongside immunomodulation with corticosteroids and IVIG reflects the standard of care. This aligns with outcomes reported by Schram AM [10], who found that combination therapy targeting the trigger and the hyperinflammation was associated with significantly improved survival in infection-associated HLH compared to immunosuppression alone [10]. The rapid clinical and laboratory improvement following this combined approach in our patient provides strong “in vivo” support for the diagnosis.

## Conclusions

This case underscores that CMV must remain a considered trigger for HLH even in immunocompetent adults presenting with fever of unknown origin and transaminitis. A low-level viremia should not dismiss the diagnosis, which relies on synthesizing clinical, laboratory, and histopathological data within the HLH-2004 framework. In addition, clinicians should include HLH on the differential when working up with FUO. As demonstrated, a timely, two-pronged therapeutic strategy - combining antiviral therapy against the trigger with immunomodulation to quell the cytokine storm - is essential to halt clinical deterioration and can be lifesaving.
